# Quantification of the Li-ion diffusion over an interface coating in all-solid-state batteries via NMR measurements

**DOI:** 10.1038/s41467-021-26190-2

**Published:** 2021-10-12

**Authors:** Ming Liu, Chao Wang, Chenglong Zhao, Eveline van der Maas, Kui Lin, Violetta A. Arszelewska, Baohua Li, Swapna Ganapathy, Marnix Wagemaker

**Affiliations:** 1 Section Storage of Electrochemical Energy, Radiation Science and Technology, Faculty of Applied Sciences, Delft University of Technology, Delft, Netherlands; 2 Key Laboratory on Power Battery Research and Shenzhen Geim Graphene Center, Tsinghua Shenzhen International Graduate School, Tsinghua University, Guangdong, 518055 China

**Keywords:** Solid-state NMR, Reaction kinetics and dynamics, Batteries, Batteries

## Abstract

A key challenge for solid-state-batteries development is to design electrode-electrolyte interfaces that combine (electro)chemical and mechanical stability with facile Li-ion transport. However, while the solid-electrolyte/electrode interfacial area should be maximized to facilitate the transport of high electrical currents on the one hand, on the other hand, this area should be minimized to reduce the parasitic interfacial reactions and promote the overall cell stability. To improve these aspects simultaneously, we report the use of an interfacial inorganic coating and the study of its impact on the local Li-ion transport over the grain boundaries. Via exchange-NMR measurements, we quantify the equilibrium between the various phases present at the interface between an S-based positive electrode and an inorganic solid-electrolyte. We also demonstrate the beneficial effect of the LiI coating on the all-solid-state cell performances, which leads to efficient sulfur activation and prevention of solid-electrolyte decomposition. Finally, we report 200 cycles with a stable capacity of around 600 mAh g^−1^ at 0.264 mA cm^−2^ for a full lab-scale cell comprising of LiI-coated Li_2_S-based cathode, Li-In alloy anode and Li_6_PS_5_Cl solid electrolyte.

## Abstract (web summary)

Development of all-solid-state batteries requires stable solid electrolyte-electrode interfaces. Here, via exchange-NMR measurements, the authors investigate the positive electrode-solid electrolyte interface, revealing the impact of an inorganic coating on the Li-ion transport properties.

## Introduction

All-solid-state lithium (Li)-ion batteries are promising candidates for next-generation high energy density and safe energy storage technology^1,2^. As a liquid-free system, it generally does not suffer from leakage and gas generation and the risk of a thermal runaway, inherent to liquid electrolytes used in conventional Li-ion batteries^3–7^. As a result, research has intensified toward solid electrolytes that display conductivities approaching, or even exceeding that of liquid electrolytes, including structural families, such as lithium superionic conductor (LISICON), argyrodites, garnets, and sodium superionic conductor (NASICON)-type structures^6,8–10^.

A major obstacle for solid-state batteries is the high internal resistance for Li^+^ transfer over the electrode-solid electrolyte interface^11,12^, which may be due to poorly conducting electrolyte decomposition products^4,13^, contact loss due to volumetric changes^4,14^ and space-charges^15,16^. The commonly applied strategy to lower the resistance for Li^+^ charge transport, is to enlarge the electrolyte-electrode interface area, often expressed as “ionic contact area”, by nanosizing the electrode and electrolyte particles^17–19^. However, with the interface area between the electrolyte and electrode, also scale the detrimental chemical and electrochemical reactions at the interfaces between the electrolyte and electrode, raising the internal resistance for Li^+^ transport. Thus, from the perspective of stability, larger electrolyte and electrolyte particles in the micron range are preferred, having the additional advantage of being more suitable for practical production^20^. However, this puts even higher demands on improving the Li^+^ transport over the relatively small ionic contact area between the micron-sized solid electrolyte-electrode particles.

To address both the stability and Li^+^ charge transport at the interfaces has lead to the development of interphases, often realized through coating processes^6,18,19^. The demands on these interphases are challenging, including (electro)chemical stability towards both electrode and electrolyte, poor electronic conductivity and at least reasonable ion conductivity, and a low grain boundary resistance with both solid electrode and electrolyte. This is typically achieved by good wettability (low interface energy) toward electrode and electrolyte and by soft interphase materials (low Young’s modules and/or yield point). One of the challenges is to assess the impact of interphases on the local Li^+^ charge transport, which can provide valuable insights for interphase design and preparation methodologies. Where the macroscopic charge transfer resistance is most often estimated by electrochemical impedance spectroscopy^21,22^, disentangling the three-phase diffusion between the electrode, interphase/coating, and electrolyte is yet to be accomplished.

One of the solid electrolyte-electrode combinations where interphase strategies are intensively investigated is sulfur-based solid electrolytes in combination with sulfur cathodes, providing a high energy density in combination with inexpensive raw materials and synthesis. Sulfide-based electrolytes (such as Li_2_S-P_2_S_5_ and Li_6_PS_5_X (X = Cl, Br, and I)) are especially promising due to their high ionic conductivity and relatively low grain boundary resistance^23–26^. These advantages, unfortunately, come along with a major drawback, which is the narrow electrochemical stability window^13,27–31^. An additional challenge is the activation of solid sulfur cathodes due to the very low Li^+^ diffusivity^32–34^, which also demands a relatively large ionic contact area^35^. To improve the Li^+^ interfacial transport two applied strategies are bilayer solid electrolytes design (porous layer and dense layer)^30,36,37^ and mixing in binary lithium halide salts (such as LiBr, LiI) additives^29–31,38–40^. Because of the large cost and sophisticated process associated with the former, halide salt addition appears especially promising for practical application. The lower internal resistance of these three-phase mixtures has been argued to be the result of the small Young’s modulus (softness) of the halide salts, which effectively act as a solid wetting agent for the electrode/electrolyte interface^29–31,38–40^. However, how the halogen salt affects the local Li^+^ transport over the grain boundaries (electrolyte-halogen salt, halogen salt-electrode, and electrolyte-electrode) is difficult to establish. This is especially important to develop fundamental understanding, guiding the optimal design and preparation of the these interphases.

Nuclear magnetic resonance (NMR) is a technique that is based on the interactions of the nuclear magnetic moment with an electromagnetic field in the radio frequency (RF) range, while a strong external magnetic field B_0_ is applied^41,42^. Recently solid-state NMR has shown the possibility of enabling selective and noninvasive measurement of Li^+^ equilibrium exchange, maintaining the dynamic equilibrium, over electrolyte-solid electrode interfaces^3,4^, which is difficult by conventional electrochemical impedance spectroscopy. This approach was further used to investigate the interfacial structure and reveal how the interface transport is influenced by the interface properties, including chemical bonds, wetting, and also space charge layers.^20,24,43,44^. In this way NMR experiments represents a powerful tool to unravel the interfacial structure and what transport process limits the performance of current solid electrolytes (SEs), which is valuable for solid electrolyte design.

Here, we explore the multiphase Li^+^ equilibrium flux between the Li_2_S electrode, LiI coating, and argyrodite Li_6_PS_5_Cl solid electrolyte in a cathodic mixture, aiming to gain insight in the role of the coating in Li-ion transport in solid-state batteries. The difference in NMR chemical shift of the three phases allows to unravel the self-diffusion between each phase via ^6^Li 2D-EXSY experiments. The sluggish Li^+^ self-diffusion over the Li_2_S-argyrodite interface is drastically improved by the presence of the LiI coating, where the Li^+^ self-diffusion is shown to proceed through the LiI coating. The activation energy between the three phases is equal to the bulk activation energy for Li^+^ diffusion in LiI, demonstrating the low grain boundary resistance achieved by the facile LiI coating strategy. The practical consequence during battery operation is that this prevents large overpotentials during battery cycling, even at a relatively low ionic contact area between the cathode and microscopic solid electrolyte particles. This is fortuitous, as it makes the working potential fall within the electrochemical stability of the argyrodite solid electrolyte. The result is a solid-state battery that can be activated at a very low applied voltage, and can cycle with high reversibility under a modest 2 MPa pressure for over 200 cycles. The ability to monitor the local Li^+^ equilibrium exchange over the grain boundaries in this three-phase system provides valuable insight in the role of coatings in achieving low interphase resistances using micron-sized solid electrolyte particles, guiding the design of stable high-performance interphases, which are crucial aspects for future solid-state batteries.

## Results and Discussion

### Stability of solid electrolytes, nanosized versus micron sized

**Fig. 1:**
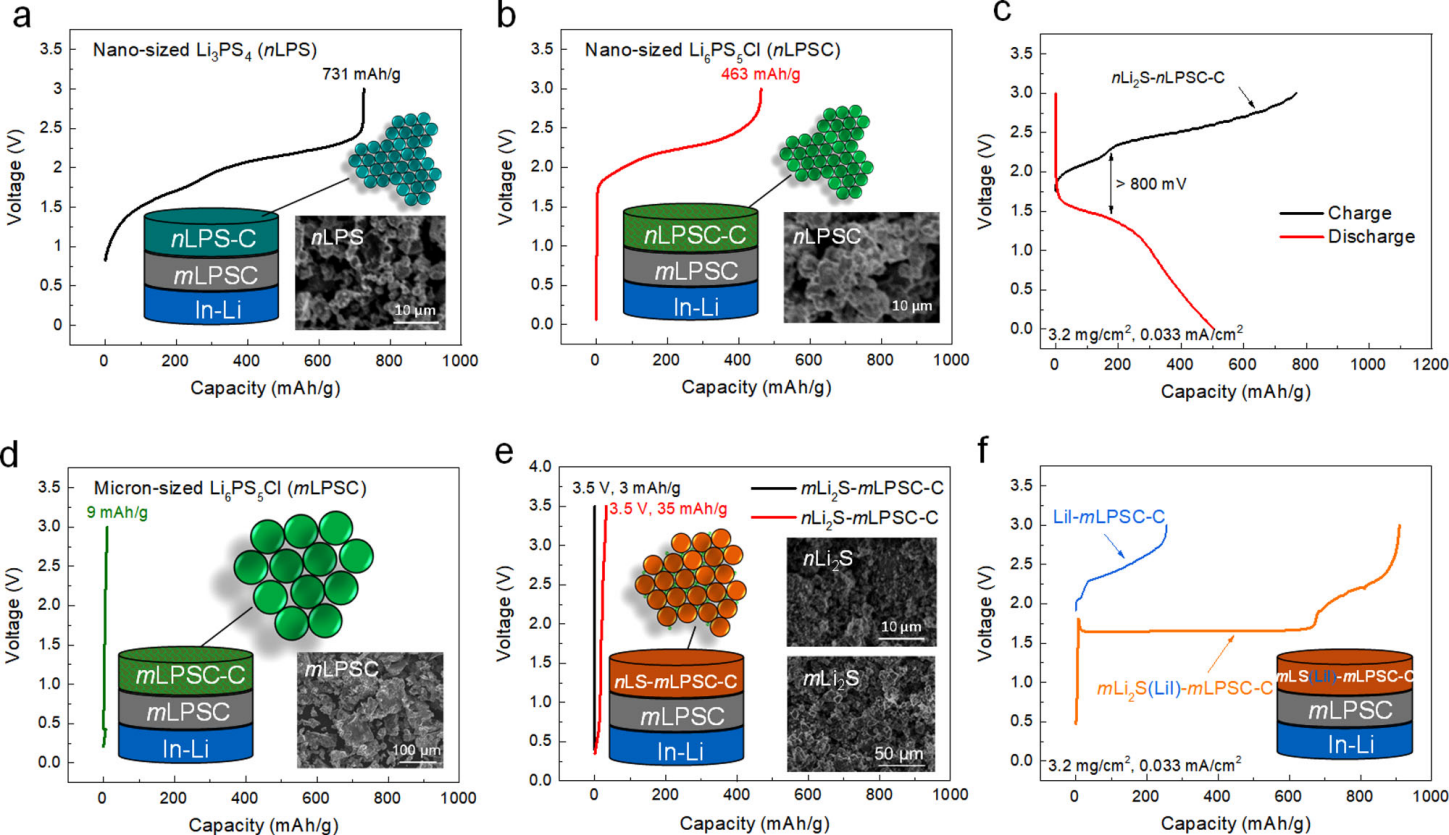
Electrochemical characterization of nano- and microsize solid-state sulfur electrolytes and sulfur-based positive electrodes. **a**, **b** voltage profiles of (**a**) nanosized *n*LPS-C and (**b**) *n*LPSC-C cathodes; In this configuration, thus by excluding the active material, the solid electrolyte acts as the active material. This is enabled by the presence of conductive carbon, providing abundant interfaces where redox reactions can occur. Therefore, this allows direct evaluation of the redox activity of the solid electrolyte that causes its decomposition. **c** Voltage profiles of *n*Li_2_S-*n*LPSC-C cathode. **d**, **e** Voltage profiles of micron-sized *m*LPSC-C, *m*Li_2_S-*m*LPSC-C, and *n*Li_2_S-*m*LPSC-C (Li_2_S is abbreviated as LS in the schematic figure). **f** Activation voltage profiles of *m*Li_2_S(LiI)-*m*LPSC-C and LiI-*m*LPSC-C cathodes; For all voltage profiles, the current density is 0.033 mA/cm^2^.

The electrochemical stability of solid electrolytes was recently shown to be significantly lower than initially thought, especially for sulfide-based solid electrolytes, where consequential decomposition reactions have been shown to have a large detrimental impact on the all-solid-state battery performance^10,45,46^. To demonstrate the redox activity of the sulfur solid electrolyte material and the impact of particle size, several mixtures of sulfur solid electrolytes with carbon are galvanostatically cycled in a home-construced lab-cell, a sketch of which is provided in Supplementary Fig. 1. As shown in Fig. 1a, b, when nanosized Li_3_PS_4_ (*n*LPS) and Li_6_PS_5_Cl (*n*LPSC) are mixed with conductive carbon (referred to as *n*LPS-C and *n*LPSC-C, respectively), they are readily oxidized at low oxidation potentials, <2 V *vs*. In (<2.62 V *vs*. Li/Li^+^), due to the sulfur S^2-^/S^0^ redox of the electrolyte, at a relatively low current density of 0.033 mA/cm^2^, in agreement with recent findings^10,25^. Thus the voltage profiles in Fig. 1a, b, represent the decomposition of the solid electrolyte, directly reflecting their electrochemical redox activity^13^. As a consequence, in a cathodic mixture these nanosized solid electrolytes will be rapidly oxidized (catalyzed by the larger interface area) in combination with common cathodes (i.e., LiCoO_2_ (LCO), LiNi_1/3_Co_1/3_Mn_1/3_O_2_ (NMC)), and thus decompose into the most stable decomposition products, which will raise the internal resistance. As a result, capacities will drop, illustrating that the decomposition of nanosized solid electrolytes in cathodic mixtures is the prime reason for the short cycle life of current all-solid-state batteries utilizing sulfide solid electrolytes^25^. The combination of a Li_2_S cathode and the LPSC electrolyte has been intensively studied^3,4,29,30^, where the harsh activation process of Li_2_S, caused by the low bulk conductivity of Li_2_S, and sluggish Li^+^ transport between the electrode and electrolyte represent another critical issue. A straightforward approach to achieve easier activation of Li_2_S and to improve the Li^+^ transport between the electrode and electrolyte is to reduce the particle size of both the Li_2_S and the LPSC (here referred to as *n*Li_2_S-*n*LPSC-C electrode). Accompanied by a large overpotential of ~800 mV, this results in a substantial capacity during the first charge and discharge and upon subsequent cycling, as shown in Fig. 1c and Supplementary Fig. 2, which is; however, hard to distinguish from the capacity of the electrolyte itself (because oxidation of LPSC is carried by the S^2-^/S^0^ redox).

The simplest strategy to reduce the contribution of the solid electrolyte to the capacity is to lower the ionic contact area through the use of micron-sized solid electrolyte particles. To verify the smaller redox activity, micron-sized LPSC (average diameter of 50 μm, as shown in Supplementary Fig. 3) was mixed with carbon, and charged (oxidized) to 3 V *vs*. In-Li (3.62 *vs*. Li/Li^+^) in the solid-state cell configuration, referred to as the *m*LPSC-C electrode. The result, shown in Fig. 1d, demonstrates nearly no contribution of the LPSC solid electrolyte to the capacity, reflecting the smaller amount of decomposition reactions under the same current density. However, as expected, the small ionic contact area of micron-sized LPSC compromises ion transport over the interface, leading to a very small capacity of the sulfur active material, even when nanosized Li_2_S is employed as shown in Fig. 1e. Although nanosized Li_2_S displays a slightly larger capacity, the rapid voltage increases to the 3.5 V vs. In-Li (4.12 V vs. Li/Li^+^) cut-off demonstrates that in both cases, the sulfur cathode material is marginally activated. At the same time, decreasing the ionic contact area increases the internal resistance and thus the overpotentials experienced at the solid electrolyte, which will induce decomposition reactions and thus driving a self-amplifying resistance growth towards battery failure.

The mechanical mixing of halogen salts like LiI with Li_2_S and sulfide solid electrolytes is an often-applied strategy, to improve conductivity, although the exact mechanism has not been clarified^29–31,38,39^. In the present study, we take a different and more controlled approach to study the detailed impact of LiI on the Li-ion transport over the grain boundaries by NMR exchange experiments, and introduce LiI at the Li_2_S-LPSC interfaces. Rather than using the conventional ball-milling route, this is achieved by introducing LiI via solution, making use of the much better solubility of LiI in ethanol compared to Li_2_S (see supporting information Supplementary Fig. 4). The solution was then evaporated at 300 ^o^C (Supplementary Fig. 4) to obtain a LiI-Li_2_S (1:3 molar ratio) composite, where LiI precipitates on the surface of Li_2_S as discussed below. This cathode was subsequently hand mixed with LPSC and C (referred to *m*Li_2_S(LiI)-*m*LPSC-C) to prepare the cathodic mixture and an all-solid-state In-Li |*m*LPSC | *m*Li_2_S(LiI) cell was assembled under 2 MPa pressure. This has a large impact on the electrochemical charging, as shown in Fig. 1f, demonstrating that the introduction of LiI results in a very low sulfur redox activation plateau at 1.69 V vs. In (2.31 V *vs*. Li/Li^+^) of the micron-sized Li_2_S combined with micron-sized LPSC. The plateau is followed by a rapid increase in potential which reflects oxidation of the solid electrolyte and/or of LiI (to LiI_3_ which is known to occur at ~2.3 V vs. In)^47,48^. To identify the contribution of the LiI and/or LPSC oxidation, this measurement was repeated in a cell without Li_2_S, shown in Fig. 1f, leading to charging at a higher voltage marking the oxidation of LPSC and/or LiI. In conclusion, deposition of LiI on Li_2_S via solution, and hand mixing this cathode with LPSC results in a low activation (oxidation) potential for Li_2_S, suggesting that facile Li^+^ transport between the electrode and electrolyte is achieved even for a relatively small ionic contact area between the micron-sized solid electrolyte and the electrode particles.

### Preparation and characterization of the LiI-coated cathode active material

**Fig. 2:**
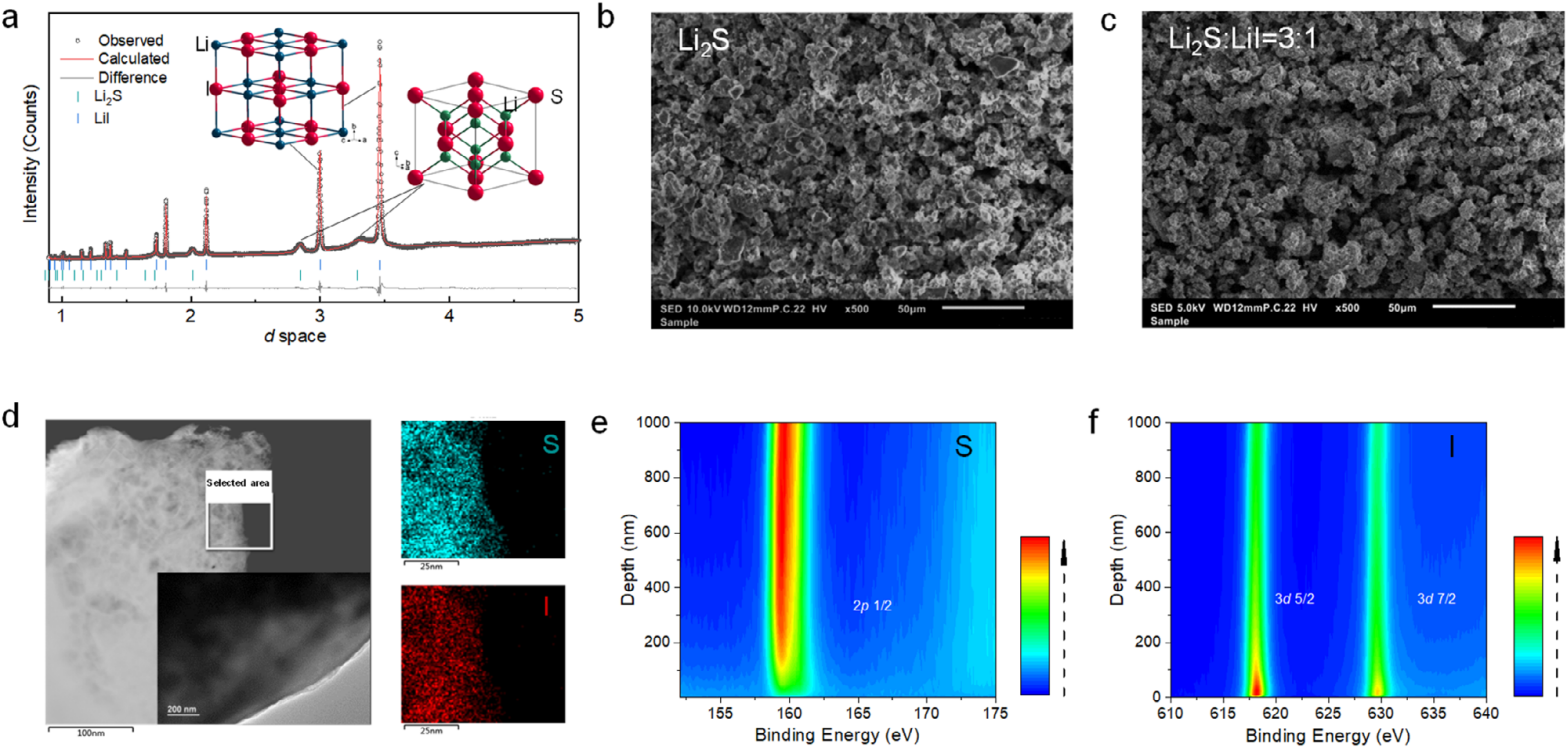
Structural and chemical characterization of the positive electrode active materials. **a** XRD pattern of the Li_2_S-LiI materials. The pattern is fit with the Rietveld method as implemented in GSAS. The inset represents the cubic structure of Li_2_S and LiI (space group of Fm3m). **b**, **c**, **d** SEM images, TEM images, and energy spectrum of the Li_2_S and Li_2_S-LiI (3:1) materials; **e**, **f** S *2p* and I *3d* XPS depth profiles of the pristine Li_2_S-LiI (3:1) materials.

To understand the role of LiI in the activation of Li_2_S, a detailed structural investigation was performed. Both the LiI and Li_2_S have a cubic structure indexed to the Fd-3m space group. Three Li_2_S-LiI composites were prepared via dissolution and precipitation where Li_2_S:LiI molar ratios of 9:1, 3:1, and 1:1 were added to ethanol, followed by evaporation of the solution at 300 °C. Li_2_S and LiI were also individually dissolved and precipitated from ethanol via evaporation for comparison. Powder X-ray diffraction (XRD) patterns of pristine Li_2_S and LiI precipitated Li_2_S and LiI and the three Li_2_S-LiI composites are provided in Fig. 2a and Supplementary Fig. 5 From a cursory inspection it can be observed that the peaks corresponding to the precipitated Li_2_S are much broader than those of the parent Li_2_S, while the peak width of precipitated LiI is comparable to the parent LiI. This indicates that on precipitation smaller primary crystallites of Li_2_S are obtained. In the three composite mixtures, both the Li_2_S and LiI phases could be indexed, albeit with shifts in peak positions of the Li_2_S component indicating changes in lattice parameters of this phase. Rietveld refinement was further performed of all the patterns depicted in Supplementary Fig. 6, and the lattice parameters obtained from the refinement are given in Supplementary Fig. 5. It can be seen that the lattice parameter of LiI (6.025 Å) remains unchanged from that of the pristine material. On the other hand, with increasing amounts of LiI in the composite the lattice parameter of Li_2_S keeps increasing from 5.701 Å (pristine) to 5.750 Å (1Li_2_S:1LiI), which might be due to the incorporation of the I^-^ in the Li_2_S lattice and/or the much smaller average crystallite size (9.18 nm for Li_2_S in 1Li_2_S:1LiI as compared to 163 nm for pristine Li_2_S).

Additional scanning electron microscope (SEM) and transmission electron microscopy (TEM) measurements are performed to study the morphology of the pristine Li_2_S and the Li_2_S-LiI mixture. As shown in Fig. 2b and c, the prepared mixture consists of a microstructure comprising of micron-sized secondary particles with a relatively uniform particle size of around 5 μm (Fig. 2c) similar to pristine Li_2_S in Fig. 2b. TEM is used to study the morphology at smaller length scales (100 nm). As seen from the TEM image and energy spectrum (Fig. 2d), the energy disperse spectroscopy (EDS) mapping of the particle surface shows uniform S and I distribution, indicating a mixture on the nanoscale was obtained with this precipitation method, and the LiI was uniformly distributed over the surface structure of Li_2_S particles. To further verify the structure of the Li_2_S-LiI material, X-ray photoelectron spectroscopy (XPS) depth profiling was performed as shown in Fig. 2e, f. The S *2p* XPS signal is relatively low until a depth of ∼100 nm, and vice versa the I *3d* is relatively high to approximately the same depth (selected window diameter is as small as 14 μm to locate only a few particles). Therefore, the present precipitation method results in micron-sized secondary cathode particles, referred to as mLi_2_S(LiI), that consist of agglomerates of LiI coated nanosized primary Li_2_S particles, where the micron-sized agglomerates are coated by a relatively thick LiI layer at some positions accumulating to form large domains of LiI (as observed with XRD).

### Investigatioun of the Li-ion diffusion mechanism

**Fig. 3:**
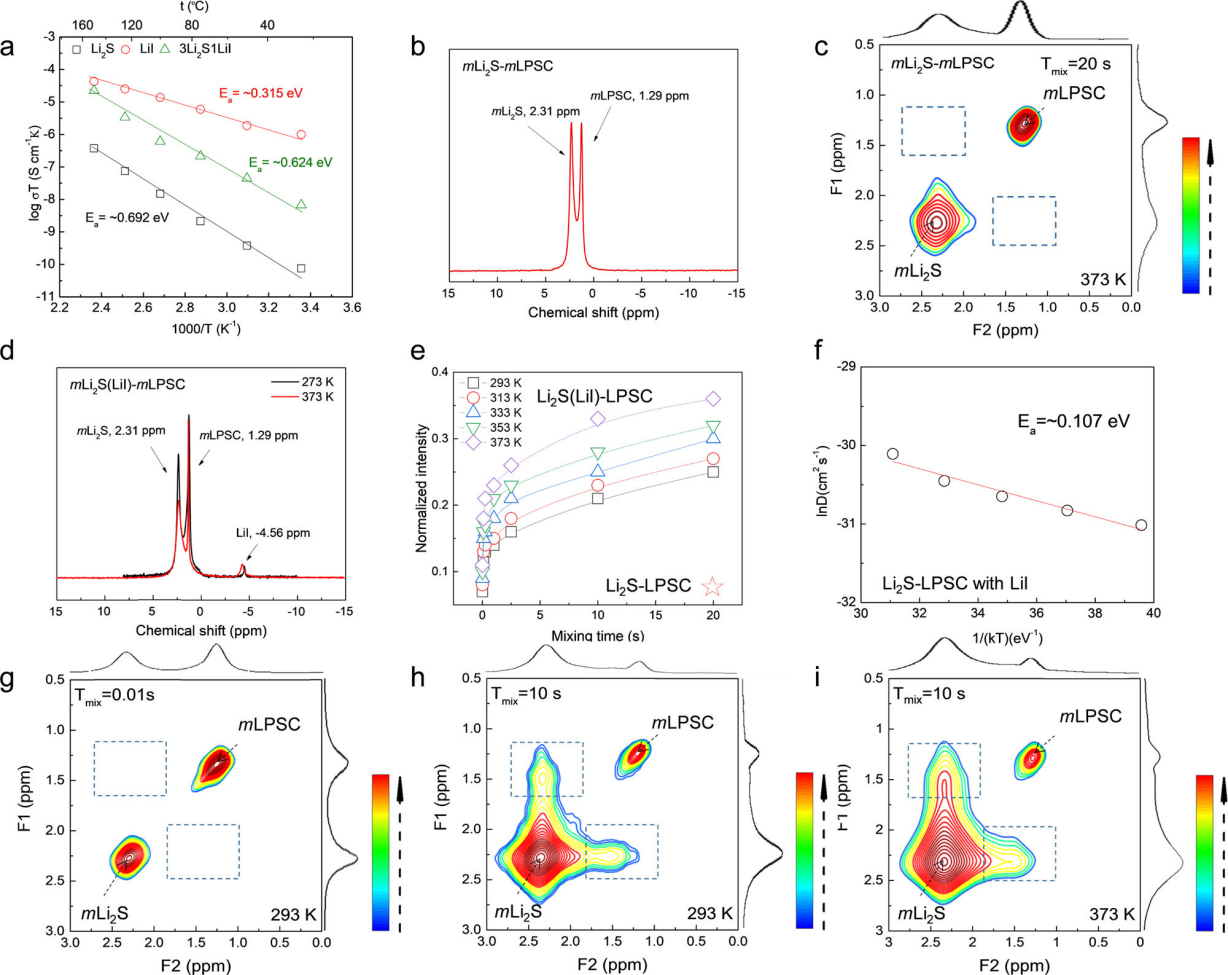
Li-ion conductivity of the positive electrode and quantification of the Li^+^ exchange across the electrode-solid electrolyte interface. **a** Ionic conductivity determined by impedance spectroscopy of pellets of Li_2_S, LiI, and the Li_2_S-LiI mixture at different temperatures. **b**, **c** One-dimensional (1D) ^6^Li magic angle spinning (MAS) and two-dimensional (2D) ^6^Li–^6^Li exchange (2D-EXSY) NMR spectra of the mLi_2_S-mLPSC cathode mixtures at a mixing time of 20 s, where Li_2_S and LPSC are both micron-sized. No obvious off-diagonal cross-peak intensity is observed, indicating that the exchange of Li^+^ over the solid–solid LPSC–Li_2_S interface is very small. **d** 1D ^6^Li MAS spectra corresponding to the mLi_2_S(LiI)-mLPSC cathode mixtures at 273 and 373 K. **e** Evolution of the cross-peak intensity as a function of T_mix_ obtained from the temperature-dependent 2D-EXSY measurements. **f** Temperature dependence of the diffusion coefficient obtained from fitting the data in (e) to a diffusion model described detail in reference^24^. These can be fit with an Arrhenius law, yielding an activation energy (E_a_) of 0.107 eV. **g–i** Two-dimensional ^6^Li-^6^Li exchange spectra of the mixture of micron-sized LPSC and Li_2_S-LiI (3:1) cathodes measured at a spinning speed of 10 kHz at (**g**, **h**) 293 K with mixing times of 0.01 s and 10 s and at (**i**) 373 K with a mixing time of 10 s. The spectra consist of 8 scans for each of the 320 slices, each slice incremented by 0.8 ms with a recycle delay of 50 s. The cross-peak at the off-diagonal positions in the dashed boxes represent the diffusion of Li-ions between solid electrolyte and electrode.

To investigate the impact of the LiI coating on the conductivity, impedance spectroscopy and ^6^Li solid NMR spectroscopy experiments are carried out. The temperature dependence of the ionic conductivities for the pristine Li_2_S and LiI as well as pellets of the 3:1 Li_2_S-LiI composite are presented in Fig. 3a. The conductivity of all the materials follow an Arrhenius law, resulting in activation energies of 0.315, 0.624, 0.692 eV for Li_2_S, LiI, and the Li_2_S-LiI mixture, respectively. The conductivity of the Li_2_S-LiI mixture (6.72 × 10^−9^ S/cm at 25 °C) is between that of Li_2_S (7.51 × 10^−11^ S/cm at 25 °C) and LiI (0.97 × 10^−7^ S/cm at 25 °C), indicating that the LiI in the Li_2_S agglomerates enhances the overall conductivity of the cathode material. To investigate the role of LiI as interphase material between the Li_2_S electrode and LPSC solid electrolyte, (2D) ^6^Li–^6^Li exchange (2D-EXSY) solid-state NMR experiments are performed. These experiments can provide selective and noninvasive quantification of the spontaneous Li^+^ diffusion, over the electrode–solid electrolyte interface (between two phases) in practical solid-state cathode mixtures, as previously reported^4,24^. This equilibrium exchange between the electrode and the electrolyte is determined by the equilibrium charge transfer kinetics between the electrode and electrolyte (the exchange current density) and the self-diffusion (diffusion in absence of a gradient in the electrochemical potential) between the two phases given the long diffusion distances. Both parameters are key indicators for the ion kinetics that determine the performance of the battery under nonequilibrium conditions. The one-dimensional (1D) ^6^Li magic angle spinning (MAS) NMR spectra of the micron-sized Li_2_S-LPSC cathode mixture, shown in Fig. 3b, displays two resonances with chemical shifts of 2.31 and 1.29 ppm, representing Li in Li_2_S and in LPSC respectively (as can be deduced from the spectra of the individual species in Supplementary Fig. 7). The difference in chemical shift between Li in Li_2_S and LPSC, that allows to distinguish both species, makes it possible to conduct the 2D exchange experiments. In the 2D exchange spectrum, Fig. 3c, both the Li^+^ environments observed in the 1D spectra are clearly observed, where the narrower LPSC resonance compared to Li_2_S is due to the higher mobility of Li^+^ in the solid electrolyte. 2D exchange-NMR effectively measures the spectrum of the ^6^Li^+^ at *t* = *0* s, then waits a mixing time *T*_*mix*_, and subsequently measure the spectrum of the same ions again at *t* = *T*_*mix*_. Li^+^ diffusion over the grain boundaries between the two chemical Li environments (Li_2_S and in LPSC) should result in off-diagonal cross-peaks, one reflecting the exchange between LPSC and Li_2_S and the other between Li_2_S and LPSC, positioned in the dotted boxes in Fig. 3c. The intensity of these cross-peaks reflects the amount of Li^+^ exchange, which is expected to increase when the diffusion time (*T*_*mix*_) and temperature are increased^4^. Note that the intensities of both cross-peaks is equal, demonstrating that the amount of Li-ions moving from the LPSC to Li_2_S is equal to that moving from the Li_2_S to the LPSC, as should be expected for equilibrium conditions where there is no net charge transfer. The absence of off-diagonal intensity, even for the maximum *T*_*mix*_ and temperature (*T*_*mix*_ = 20 s, 373 K) indicates that the Li^+^ exchange (flux) over the solid–solid interface between LPSC and Li_2_S (without LiI coating), is too small to be observed, reflecting sluggish Li^+^ mobility across the interface with the solid electrolyte. This rationalizes the observation in Fig. 1d, that these mixtures do not facilitate activation of Li_2_S. As expected, the addition of LiI to the cathodic mixture, *m*Li_2_S(LiI)-*m*LPSC, results in the appearance of the Li resonance at −4.56 ppm associated with LiI (referenced to Supplementary Fig. 7), in the 1D ^6^Li NMR spectrum (Fig. 3d). The impact of the LiI on the spontaneous Li^+^ exchange, between the Li_2_S and LPSC is dramatic, as can be observed in Fig. 3e–i. At short mixing time, *T*_*mix*_ = 10 ms, no appreciable cross-peak intensity is observed in the 2D-EXSY spectrum (Fig. 3g). However, increasing the mixing time, *T*_*mix*_, to 10 s, and raising the temperature to 373 K, results in a strong cross-peak intensity (Fig. 3h, i), which is a measure of the Li^+^ exchange between Li_2_S and LPSC. It should be stressed that (Fig. 3h, i) the off-diagonal intensity is not perfectly centered at the expected cross-peak positions between Li_2_S (2.31 ppm) and LPSC (1.29 ppm). The misalignment with the main Li_2_S resonance appears to be due to Li-ion environments at a slightly smaller chemical shift (2.27 ppm) compared to the main Li_2_S peak. Indeed, the Li_2_S resonance is assymetric, more clearly observed in the cross-section of the 2D-EXSY spectrum (Supplementary Fig. 8a). We suggest that this represents the LiI rich Li_2_S-LiI mixtures at the surface of the Li_2_S particles, which will exchange Li-ions with LPSC on a shorter timescale because of the smaller diffusion distance, based on the missallignment in Fig. 3g. Raising the temperature decreases the missallignment, as observed in Fig. 3i, indicating that Li-ion exchange with the bulk Li_2_S, having a longer diffusion pathway to LPSC, is activated. The misalignment of the crosspeak with the main LPSC resonance appears to be due to the two Li-environments present in the LPSC spectrum. Micron-sized LPSC displays two Li-environments at 1.44 and 1.29 ppm as observed from the 1D ^6^Li NMR spectrum of pristine, as synthesized micron-sized LPSC (Supplementary Fig. 8b). This is also resolved in the cross-sections at 1.44 ppm of the 2D spectra as Supplementary Fig. 8a. The assignment of the two resonances is unclear at this stage, but we speculate this is due to heterogeneity in the distribution of S and Cl on the 4a and 4c sites in LPSC particles arising during synthesis, which is known to affect the conductivity^23^. The missallignment of the crosspeak indicates that the Li-exchange is dominated by the shoulder at 1.44 ppm, suggesting that this is the more conductive LPSC environment. The evolution of the normalized cross-peak intensity as a function of *T*_*mix*_ measured at a range of temperatures and a *T*_*mix*_ range of 10 ms−10 s is provided in Fig. 3e. The exchange between the Li_2_S and LPSC phases was further quantified by fitting the evolution of the cross-peak intensity as a function of *T*_*mix*_ to a diffusion model derived from Fick’s law, described elsewhere^24^. From the fit, the diffusion coefficient (D) as a function of temperature can be obtained, which in this case pertains to Li-ion transport across the Li_2_S-LPSC interface. The diffusion coefficients as a function of temperature obtained from the fit are given in Fig. 3f. The data for Li_2_S-LPSC diffusion can be fit to an Arrhenius law, yielding an activation energy of 0.107 eV for the Li^+^ transfer. The value for the activation energy and observed Li^+^ exchange is comparable to that obtained for interfacial diffusion between nanosized Li_2_S and nanosized (average particle size ~100 nm) LPSC argyrodite (0.10–0.13 eV)^3,4^. The conclusion is that, despite the small ionic contact area of the present micron-sized LPSC (average particle size ~50 μm) in the cathodic mixtures, the interface transport is improved to such an extent that it matches that of nanostructured mixtures having a much large ionic contact area. The two orders of magnitude difference in diameter between the LPSC in the cathodic mixtures, suggests that the LiI improves the Li^+^ diffusion over the interface with four orders of magnitude.

**Fig. 4:**
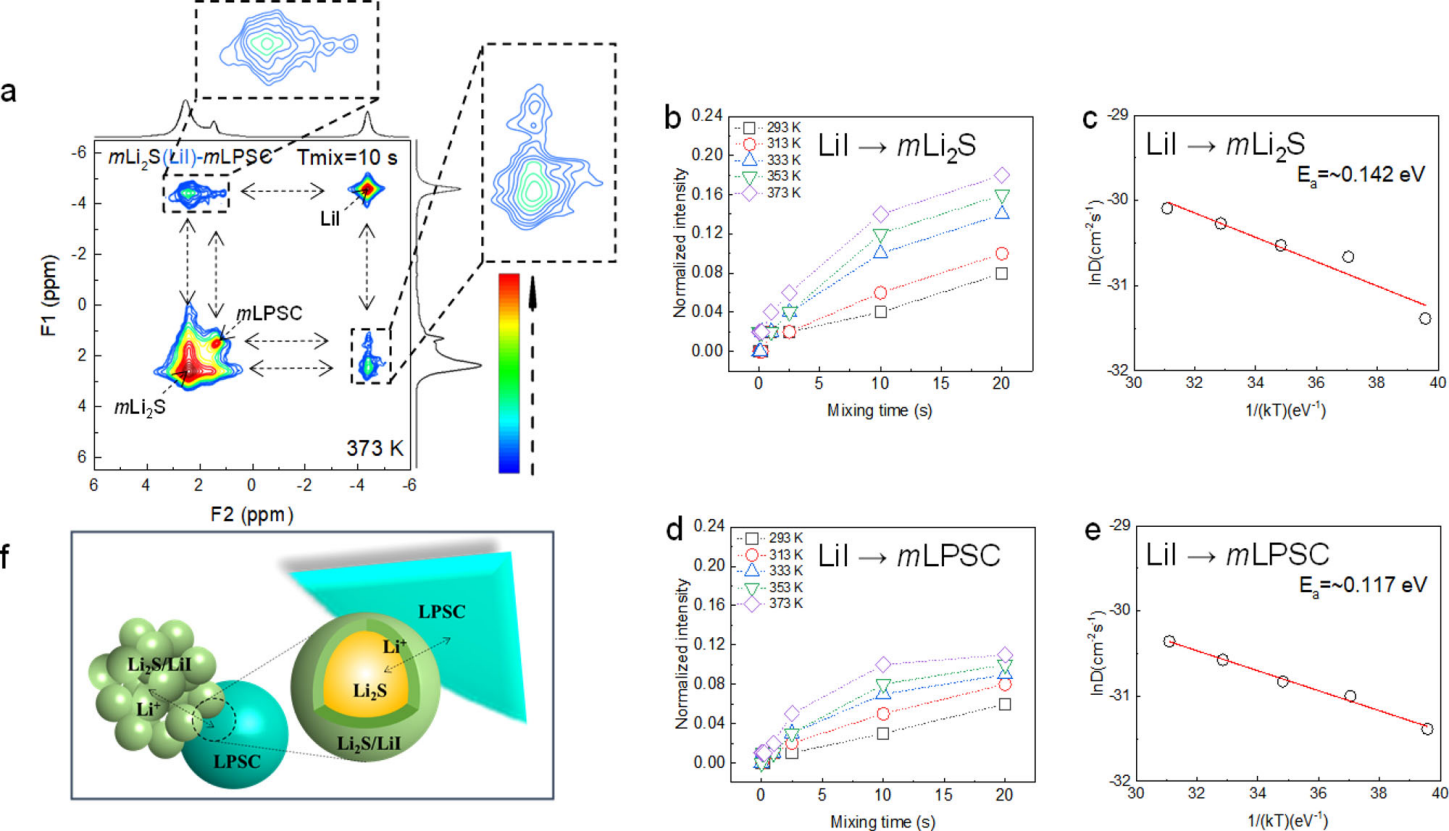
Mechanism for Li^+^ transport in the *m*Li_2_S(LiI)-*m*LPSC cathodic mixtures. **a** Two-dimensional ^6^Li-^6^Li exchange spectra of the mixture of LPSC and Li_2_S-LiI powders measured at spinning speed of 10 kHz at 100 °C and a mixing time of 10 s. **b, c** Evolution of the cross-peak intensity as a function of T_mix_ obtained from the temperature-dependent 2D-EXSY measurements. **d**, **e** Temperature dependence of the diffusion coefficient obtained from fitting the data in (**b**, **d**). **f** Schematic of the proposed Li^+^ transport mechanism in the mLi_2_S(LiI)-mLPSC-C cathodic mixtures.

To understand the role of the LiI in the diffusion, Fig. 4a focuses on the exchange of Li in Li_2_S and LPSC with LiI, hence the three-phase Li-ion exchange in the 2D-EXSY measurements shown in Fig. 3. Fig. 4a displays a clear exchange of Li^+^ between LiI and both Li_2_S and LPSC, reflecting the equilibrium exchange of Li^+^ between the three phases in the cathodic mixture. This represents a unique view into the Li^+^ diffusion between the coating and the electrode and electrolyte phases in a solid-state battery. By measuring and fitting the exchange intensities as a function of mixing time and temperature, Fig. 4b-e, similar to the evaluation of the direct exchange between the Li_2_S and LPSC, the diffusion coefficients and activation energy over both the LPSC-LiI and LiI-Li_2_S interfaces is quantified. To the best of our knowledge, this is the first quantification of the local ion diffusion between a coating and its facing solid phases, providing insight in the impact of a coating on the Li-ion transport. The high diffusivity and very low activation energies for Li^+^ transfer from Li_2_S to LiI and LPSC to LiI, 0.142 eV and 0.117 eV respectively, are similar to the overall Li^+^ transfer between Li_2_S and LPSC. This indicates that LiI facilitates the Li-ion exchange and thus functions as the bridge between electrode and electrolyte as summarized schematically in Fig. 4f. Apparently, the ductile LiI^40^ creates grain boundaries between both the electrolyte and electrode material that do not pose an additional barrier for Li-ion diffusion, and the diffusivity of LiI itself dictates the diffusivity between electrode and electrolyte.

### Electrochemical performance

**Fig. 5:**
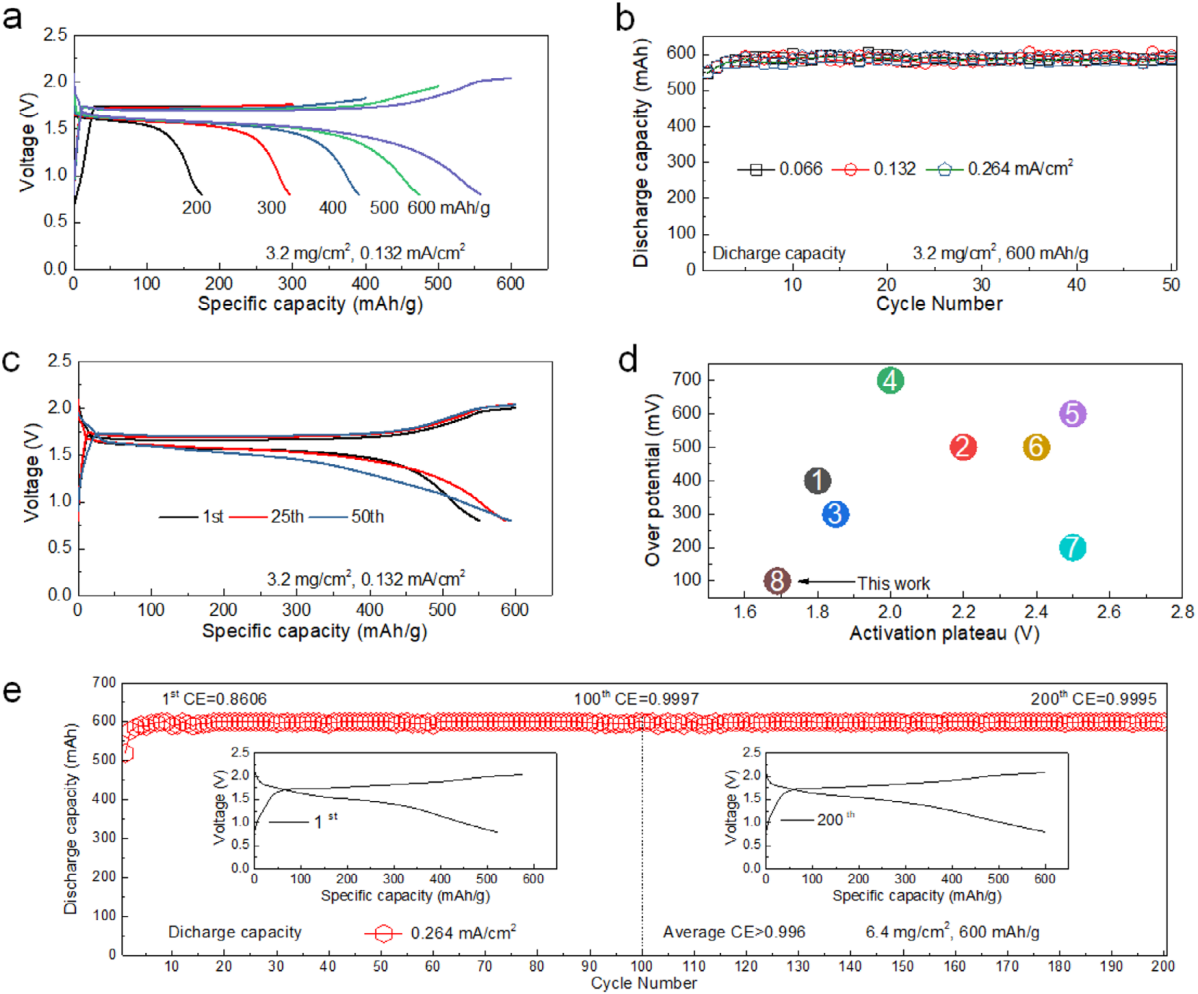
Electrochemical energy storage performances of a In-Li | *m*LPSC | *m*Li_2_S(LiI) cell. **a** Charge and discharge curves of the In-Li | *m*LPSC | *m*Li_2_S(LiI) cell cycled incrementally from 200 to 600 mAh/g at a 0.132 mA/cm^2^ current density; **b** Discharged capacity of the In-Li | *m*LPSC | *m*Li_2_S(LiI) cell cycled to 600 mAh/g at 0.066, 0.132 and 0.264 mA/cm^2^ respectively; **c** 1st, 25th, and 50th charge and discharge curves of the In-Li | *m*LPSC | *m*Li_2_S(LiI) cell cycled at 0.132 mA/cm^2^ current density; **d** Overpotential versus average activation voltage plateau comparing results from literature as listed in Supplementary Table 1 with the present work; **e** Discharge capacity of the In-Li |*m*LPSC | *m*Li_2_S(LiI) cell cycled to 600 mAh/g at 0.264 mA/cm^2^ current density up to 200 cycles. The discharged capacity divided by 600 is identical to the Coulombic efficiency, because the cells were charged to a fixed capacity of 600 mAh/g.

To test the efficacy of the Li_2_S-LiI cathode in combination with the micron-sized LPSC, In-Li | *m*LPSC | *m*Li_2_S(LiI), all-solid-state cells were assembled, during which only a pressure, 2 MPa, was applied and removed afterwards (see methods section for assembly details). The cell performance is shown in Fig. 5. Although the cell can be activated during charge to over 900 mAh/g capacity as shown in Fig. 1e, discharge leads to large overpotentials. The increase of the oxidation potential towards 900 mAh/g exceeds the oxidation potential of LiI (2.3 V vs. In-Li) and that of the LPSC electrolyte (2.1 V vs. In-Li), which will result in poorly conducting species near the interfaces that increase the impedance. To prevent this, the cell is charged to specific capacities (fixed charge capacity) as shown in Fig. 5a, followed by discharging to a fixed potential (0.8 V vs. In-Li) to achieve complete discharge. After the cell is initially charged to 600 mAh/g directly, the cell was cycled up to 50 cycles at different currents with an average Coulombic efficiency higher than 97.8% (Fig. 5b). The 1st, 25th, and 50th charge and discharge curves of the In-Li | *m*LPSC | *m*Li_2_S(LiI) cell cycled at 0.132 mA/cm^2^, shown in Fig. 5c, demonstrate an ultra-low activation potential of 1.7 V *vs*. In-Li, amounting an overpotential of only ~100 mV. This is the lowest overpotential reported (Fig. 5d and Supplementary Table 1) to date and the consequence of the facile Li-ion transport induced by the LiI coating as observed by the exchange-NMR experiments. Post-mortem XRD analysis after different states of charge, and NMR analysis after 50 cycles (ending in the charged state) of the cycled *m*Li_2_S(LiI)-*m*LPSC-C active materials can be found in Supplementary Fig. 9. After the first charge to 600 mAh/g and even after the 50^th^ charge to 600 mAh/g, only the Li_2_S is oxidized, towards an amorphous structure, and the micron-sized LPSC solid electrolyte remains intact as no decomposition products are observed^49^. The long cycling stability of the cell with a higher mass loading, 6.4 mg/cm^2^, is demonstrated in Fig. 5e. After 200 cycles, the Coulombic efficiency maintains values exceeding 99.9% resulting in an average Coulombic efficiency of 99.6%. Most notable is that this is achieved in combination with micron-sized electrolyte particles, a small ionic contact area, and that further optimization can be expected upon variation of the amount of LiI, the applied pressure, charge capacity, and the cathodic mixture.

In summary, the impact of a ductile coating on the diffusion over the grain boundaries between the electrode and solid electrolyte in a cathodic mixture is investigated. Solid-state ^6^Li NMR is able to distinguish between the Li environments in the Li_2_S electrode, LiI coating, and argyrodite Li_6_PS_5_Cl solid electrolyte. This enables 2D exchange-NMR measurements between these environments, which allows quantification of the equilibrium exchange of Li-ions, driven by self-diffusion, between the electrode, coating, and solid electrolyte. In this manner, the impact of the coating on the Li^+^ diffusion can be evaluated. Effectively, the ductile LiI lowers the barrier for grain boundary diffusion towards both the Li_2_S (electrode) and Li_6_PS_5_Cl (electrolyte) phases to such an extent that the conductivity of the thin LiI coating dominates. This improves the Li-ion exchange between the electrode and electrolyte with several orders of magnitude and enables to move from nanostructured solid-state cathode mixtures to micron-sized solid-state cathode mixtures, the latter having the practical advantages of high stability and facile material and electrode production. The impact of the improved Li-ion exchange is demonstrated by a sulfide-based solid-state battery which combines easy activation of the sulfur electrode at very low overpotentials with stable cycling. This work demonstrates the ability of exchange-NMR unambiguously quantify and disentangle the Li^+^ diffusion over the interfaces between electrode, coating, and solid electrolyte (three-phase exchange) in solid-state batteries. As one of the key challenges towards solid-state batteries is the development of interphases to establish stability and facile Li^+^ transport, the present approach and insights provides valuable insights to guide future understanding and material design.

## Methods

### Solid-electrolyte and cathode active materials preparation

The solid-state electrolyte Li_6_PS_5_Cl (denoted as LPSC) was prepared by a simple solid-state reaction. The stoichiometric raw materials LiCl (Sigma-Aldrich), P_2_S_5_ (Sigma-Aldrich), and Li_2_S (Sigma-Aldrich) were used as the starting materials and were ball milled at 110 rpm, 2 h with the ZrO_2_ coated jars using 18 ZrO_2_ balls. After the ball milling, the precursor was sealed in a quartz tube with Ar and then annealed at 550 ^o^C for 15 hours to obtain the LPSC solid electrolyte. These were subsequently crushed with an agate mortar-pestle before using the samples for further measurements. For preparation of the LiI-coated Li_2_S cathode active material, proportional 10 mmol Li_2_S and 3.33 mmol LiI were dissolved into 1 ml ethanol and stirred for 10 mins then heated at 300 ^o^C until totally dry.

### Material characterization

XRD patterns were collected over a two-theta range of 10–80° to identify the crystalline phases of the prepared materials using CuKα X-rays (1.5406 Å at 45 kV and 40 mA) on an X’Pert Pro X-ray diffractometer (PANalytical). To prevent reaction with moisture and oxygen, the powder materials were sealed in an airtight XRD sample holder in an argon-filled glove box. For the TEM and energy dispersive X-ray (STEM-EDX) investigations, a suspension in dry ethanol was prepared, which was drop casted onto a standard gold grid with a holy carbon film under a high temperature, inside an argon-filled glove box. To prevent any contact with air TEM grids with the sample were loaded into a custom-made vacuum transfer TEM holder. TEM measurements were carried out in a FEI-Tecnai operating at 200 kV. Field emission (FE)-SEM (JEOL JSM-6010LA) images were taken under dry Argon conditions, and taken using an accelerating voltage of 10 kV. The depth-profiling sputtering was conducted by 2 min of sputtering in five cycles (2 kV, 2 mm × 2 mm), the narrow spectra of particular elements were recorded after each cycle of sputtering. The pass energy used for the hemispheric analyzer was 58.7 eV, and the base pressure of the system was ∼1 × 10^−7^ Pa. The estimated sputtering rates are 5 nm/min. The ionic conductivities were measured by electrochemical impedance spectroscopy (EIS) where 200 mg of the material is pressed into a pellet at 2 Mpa and assembled in the all-solid-state cell as described below. The pellet’s are kept at each test temperature (from 25 to 150 °C) for at least half an hour before the electrochemical impedance measurements were acquired, in order to reach thermal equilibrium. The EIS measurements were carried out from 100 kHz to 1 Hz with an alternating current amplitude of 5 mV using an Autolab (PGSTAT302N).

### All-solid-state cell assembly and electrochemical testing

Solid-state cells were assembled in an argon-filled glove box (O_2_ < 0.1 ppm, H_2_O < 0.1 ppm) in the following steps: The positive electrode was prepared by hand-mixing the cathodic mixture with ratio of LiI-coated Li_2_S active material, solid electrolyte and carbon additive (Super-P) of 4: 4: 2. 150 mg of LPSC was pressed at 0.5 Mpa as the electrolyte layer, and subsequently ~12 mg of cathodic mixture and a In-Li foil were brought into contact to the top and bottom faces of the solid electrolyte pellet. A 9 mm in diameter In film was placed between the LPSC pellet and a 6 mm Li metal film (MTI-KJ group) during cell assembly, where no processing of the metal was carried out during cell assembly. After that, 2 MPa pressure (press machine) was applied during 30 s and removed afterwards, to establish contact between the three layers. The thickness of the electrode and electrolyte is ~150 and ~1200 μm, respectively, and the diameter of both electrode and electrolyte is 10 mm. The cell used for testing is a home-construced lab-cell, a sketch of which is provided in Supplementary Fig. 1. The galvanostatic cycling experiments were performed with a programmable Maccor 4000 series galvanostat. The cells were charged to a fixed specific capacity (200–600 mAh/g) and then discharged to 0.8 V vs In-Li at various current densities (0.066–0.264 mA/cm^2^). The electrochemical experiments were carried out at a constant room temperature of 24 °C.

### Solid-state ^6^Li NMR measurements

Solid-state NMR measurements were performed on a Bruker Ascend 500 spectrometer (**B**_**0**_ = 11.7 T) with a NEO console operating at a ^6^Li resonance frequency of 73.578 MHz. Chemical shifts were referenced with respect to a 1 M LiCl solution. A Bruker two-channel MAS WVT 4 mm probe was used for all measurements. Single-pulse ^6^Li experiments were performed with π/2 pulse lengths of 4.90 μs. A recycle delay of three times of T_1_ was used each time, where T_1_ was determined using saturation recovery experiments. Two-dimensional exchange spectroscopy (2D-EXSY) measurements were performed for the mixture at a spinning speed of 10 kHz and at various mixing times ranging from 10 ms up to 20 s and at temperatures from 20 to 100 °C. It should be noted that the duration of this “mixing” period, T_mix_, is limited only by the nuclear spin’s longitudinal relaxation time T_1_. Due to the long T_1_ of ^6^Li, a smaller spectral width of 1250 Hz in F1 was used. All 2D spectra were measured with of 8 scans for each of the ~320 transients, each transient incremented by 0.8 ms with a recycle delay of up to 50 s, depending on the temperature. The exchange between the Li_2_S and LPSC phases was quantified by fitting the evolution of the cross-peak intensity as a function of T_mix_ to a 3D diffusion model derived from Fick’s law^4,24^.

## Supplementary information

Supplementary Information

## Data Availability

The data that support the findings within this paper are available from the corresponding author on request.
