# Author Correction: Quantification of the Li-ion diffusion over an interface coating in all-solid-state batteries via NMR measurements

**DOI:** 10.1038/s41467-023-38112-5

**Published:** 2023-04-25

**Authors:** Ming Liu, Chao Wang, Chenglong Zhao, Eveline van der Maas, Kui Lin, Violetta A. Arszelewska, Baohua Li, Swapna Ganapathy, Marnix Wagemaker

**Affiliations:** 1 Section Storage of Electrochemical Energy, Radiation Science and Technology, Faculty of Applied Sciences, Delft University of Technology, Delft, Netherlands; 2 Key Laboratory on Power Battery Research and Shenzhen Geim Graphene Center, Tsinghua Shenzhen International Graduate School, Tsinghua University, Guangdong, 518055 China

**Keywords:** Solid-state NMR, Reaction kinetics and dynamics, Batteries, Batteries

Correction to: *Nature Communications* 10.1038/s41467-021-26190-2, published online 12 October 2021

The original version of this article contained errors in Figure 3a and Figure 3f. In Figure 3a, the activation energies (Ea) were calculated using a log scale instead of a logarithm ln scale. In Figure 3f, the y-axis interval was not properly selected. The correct y-axis interval in Figure 3f and the numerical values of the activation energy are now provided in Figure 3a and the main text. These errors have been corrected in the HTML and PDF versions of the article.

