# Supplementary material for: Quantification of the Li-ion diffusion over an interface coating in all-solid-state batteries via NMR measurements
Source: Nat Commun. 2021 Oct 12;12:5943. doi: 10.1038/s41467-021-26190-2 (PMC8511027; doi:10.1038/s41467-021-26190-2)
Supplement: Supplementary file 1 — Supplementary Information [file 41467_2021_26190_MOESM1_ESM.pdf]

Supplementary information

Quantification of the Li-ion diffusion over an interface coating in all-solid-state batteries via NMR measurements

Ming Liu[1], Chao Wang[1], Chenglong Zhao[1,2], Eveline van der Maas[1], Kui Lin[2], Violetta A. Arszelewska[1], Baohua Li[2], Swapna Ganapathy[1*] and Marnix Wagemaker[1*]

[1] Section Storage of Electrochemical Energy, Radiation Science and Technology, Faculty of Applied Sciences, Delft University of Technology. E-mail: s.ganapathy@tudelft.nl and m.wagemaker@tudelft.nl

[2] Key Laboratory on Power Battery Research and Shenzhen Geim Graphene Center, Tsinghua Shenzhen International Graduate School, Tsinghua University, Guangdong 518055, China.

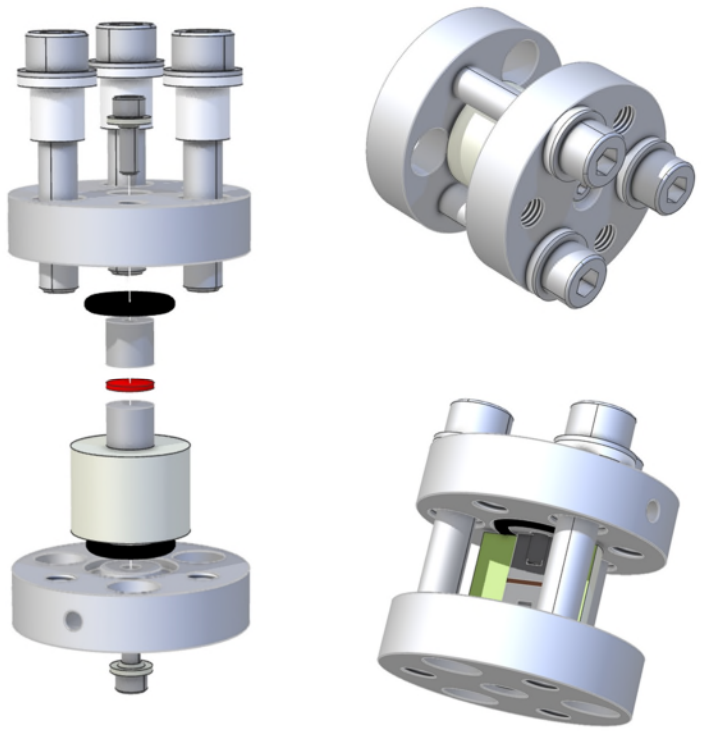

Supplementary Figure 1| Schematic drawing of the lab-scale cell used for the electrochemical energy storage experiments.

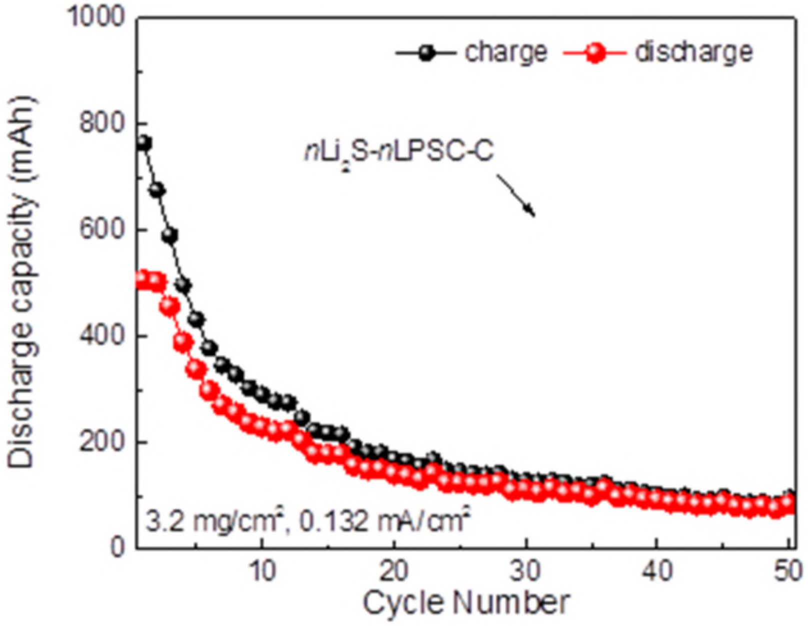

Supplementary Figure 2| Cycle performance of a cell with the *n*Li_2S-*n*LPSC-C cathodic mixture as assembled in the methods section.

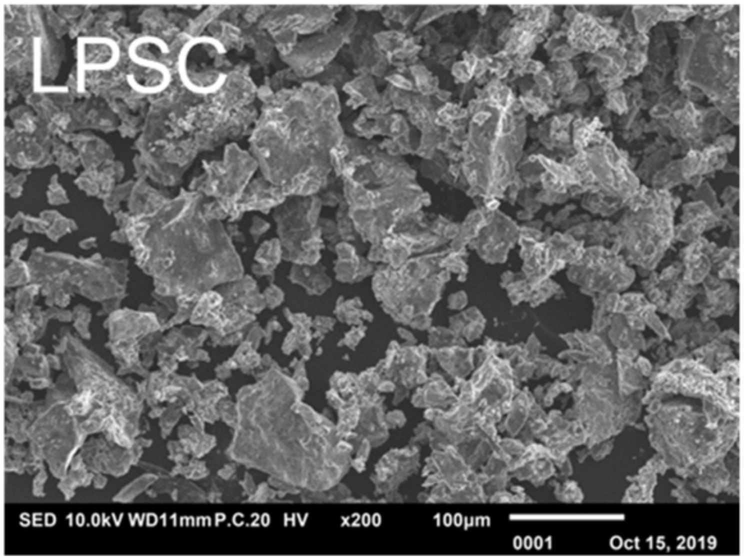

Supplementary Figure 3| SEM images of pristine micron sized Li_6PS_5Cl (LPSC).

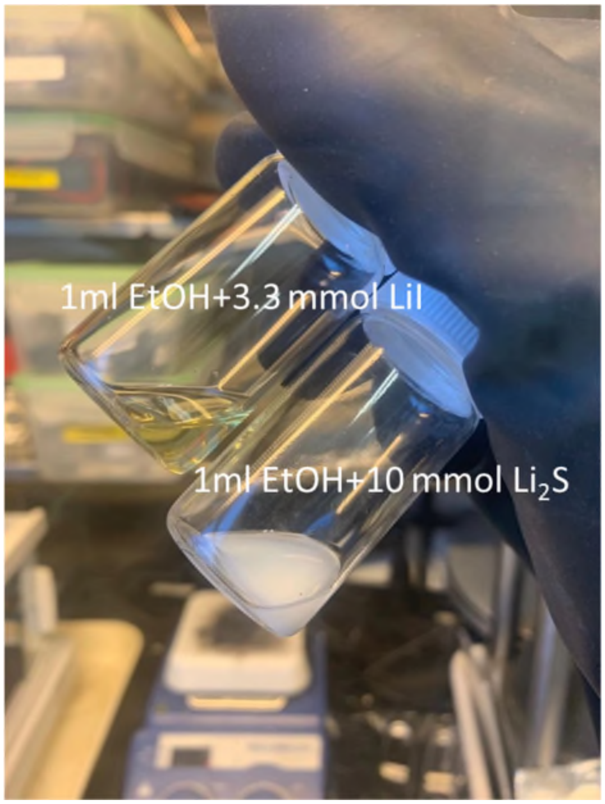

Supplementary Figure 4| Optical image of 3.3 mmol fully dissolved LiI and 10 mmol partially dissolved Li_2S in 1 ml ethanol, respectively.

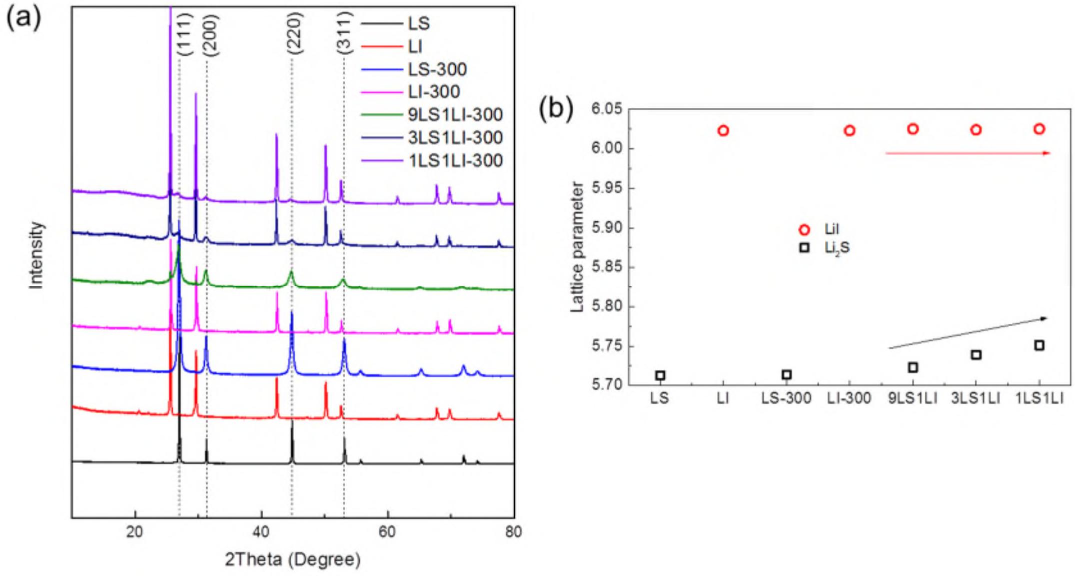

Supplementary Figure 5| (a) XRD patterns and (b) lattice parameters of the Li_2S, LiI and Li_2S-LiI materials. All the patterns are fit with the Rietveld method as implemented in GSAS.

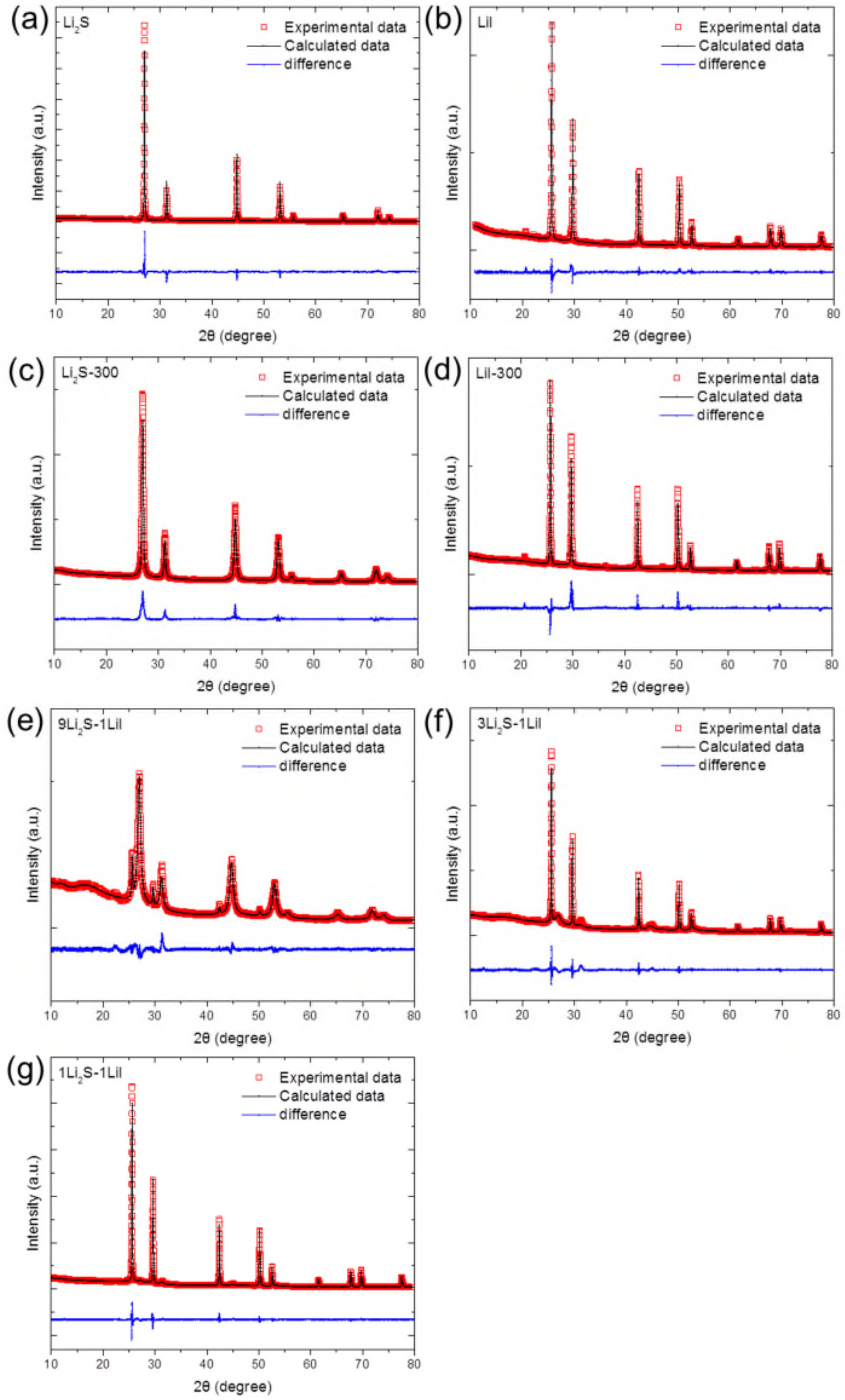

Supplementary Figure 6| Rietveld refinement of Li_2S, LiI and Li_2S-LiI based on the diffraction patterns given in **Supplementary Figure 5**.

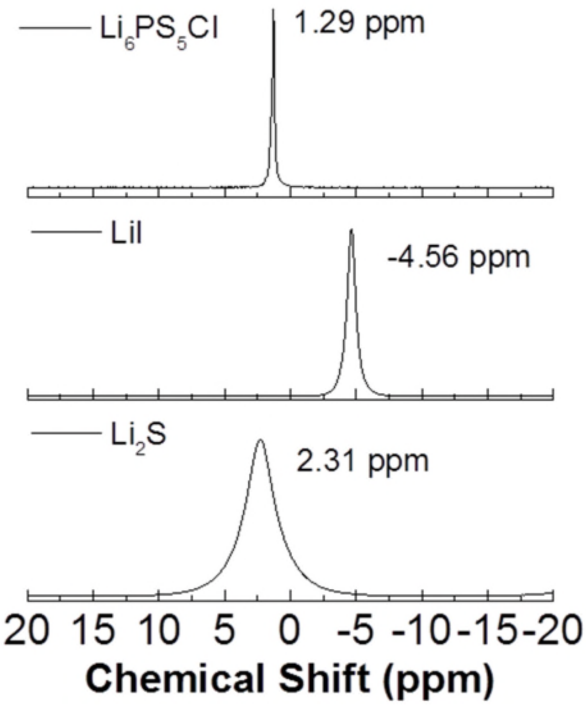

Supplementary Figure 7| One-dimensional (1D) ^{6}Li magic angle spinning (MAS) spectra of the individual Li_2S, LiI and Li_6PS_5Cl materials.

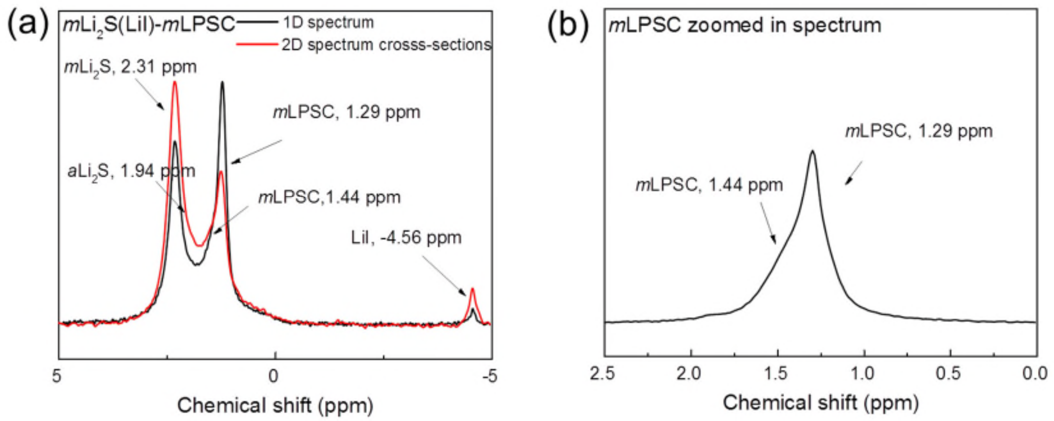

Supplementary Figure 8| (a) 1D ^{6}Li MAS spectrum and 2D EXSY cross sections of Li_2S-LiI-Li_6PS_5Cl mixtures (*a*Li_2S for asymetry of the Li_2S peak), (b) Zoomed in spectrum of *m*LPSC.

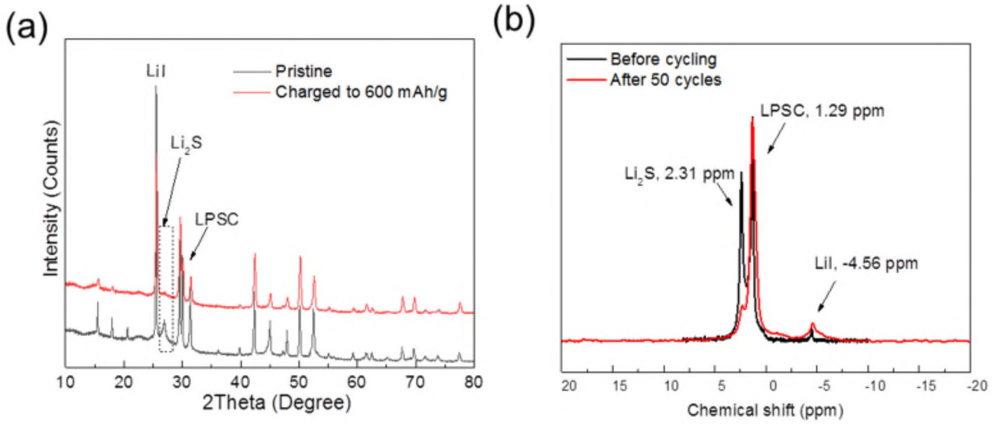

Supplementary Figure 9| Post mortem (a) XRD and (b) NMR analysis of the *m*Li_2S(LiI)-*m*LPSC-C active materials after charging to 600 mAh/g and after 50 cycles to 600 mAh/g (disassembled at the state of charging the cell to 600 mAh/g, and then measured the cathode mixture).

Supplementary Table 1| Activation voltage and over potential in our work compared with open literature as shown in **Figure 4d**.

Cell Configuration	Solid electrolyte content in the cathode	Temperature	Pressure during measuring the battery	Activation plateau	Over-potential	Reference number
In-Li | LPSC | Li_2S	40%	RT	n/a	~1.8 V vs. In-Li	~400 mV	1
In-Li | SE | Li_2S-LiI	n/a	RT of 25 ^{0}C	72 Mpa	~2.4 V vs. In-Li	~500 mV	2
In-Li | $Li_7P_3S_{11}$ | Li_2S@C	42%	60 ^{0}C	n/a	~1.85 V vs. In-Li	~300 mV	3
In-Li | LPSC | Li_2S	30%	RT	n/a	~2 V vs. In-Li	~700 mV	4
In-Li | Li_2S-P_2S_5 | **Li_2S-VGCF**	58%	RT of 25 ^{0}C.	Coin cell pressure	~2.5 V vs. In-Li	~600 mV	5
**Li | LPS–Kevlar| Li_2S-LiI–VGCF–SS**	15%	RT	n/a	~2.4 V vs. In-Li	~500 mV	6
In-Li | Li_2S-P_2S_5 | carbon coated Li_2S	62%	RT	Coin cell pressure	~2.5 V vs. In-Li	~200 mV	7
In-Li | *m*LPSC | LiI coated *m*Li_2S	40%	RT of 24 ^{0}C.	~2 Mpa	1.69 V vs. In-Li	~100 mV	This work

References

1 Yu, C. *et al.* Accessing the bottleneck in all-solid state batteries, lithium-ion transport over the solid-electrolyte-electrode interface. *Nat Commun* **8**, 1086 (2017).

2 Hakari, T. *et al*. Li_2S-Based Solid Solutions as Positive Electrodes with Full Utilization and Superlong Cycle Life in All-Solid-State Li/S Batteries. *Adv Sustain Syst* 1, 1700017 (2017).

3 Yan, H. F. *et al.* In Situ Generated Li_2S-C Nanocomposite for High-Capacity and Long-Life All-Solid-State Lithium Sulfur Batteries with Ultrahigh Areal Mass Loading. *Nano Lett* **19**, 3280-3287 (2019).

4 Han, F. *et al.* High-Performance All-Solid-State Lithium-Sulfur Battery Enabled by a Mixed-Conductive Li_2S Nanocomposite. *Nano Lett* **16**, 4521-4527, (2016).

5 Eom, M. *et al.* High performance all-solid-state lithium-sulfur battery using a Li_2S-VGCF nanocomposite. *Electrochimica Acta*, **230**, 279-284 (2017).

6 Xu, R. C. *et al.* Cathode-Supported All-Solid-State Lithium-Sulfur Batteries with High Cell-Level Energy Density. *Acs Energy Lett* **4**, 1073-1079 (2019).

7 Choi, S.et al. Carbon-coated Li_2S cathode for improving the electrochemical properties of an all-solid-state lithium-sulfur battery using Li_2S-P_2S_5 solid electrolyte. *Ceramics International* **44**, 7450-7453 (2018).
